# ^64^Cu^2+^ Complexes of Tripodal Amine Ligands’ In Vivo Tumor and Liver Uptakes and Intracellular Cu Distribution in the Extrahepatic Bile Duct Carcinoma Cell Line TFK-1: A Basic Comparative Study

**DOI:** 10.3390/ph17070820

**Published:** 2024-06-21

**Authors:** Mitsuhiro Shinada, Masashi Takahashi, Chika Igarashi, Hiroki Matsumoto, Fukiko Hihara, Tomoko Tachibana, Masakazu Oikawa, Hisashi Suzuki, Ming-Rong Zhang, Tatsuya Higashi, Hiroaki Kurihara, Yukie Yoshii, Yoshihiro Doi

**Affiliations:** 1 Faculty of Science, Toho University, Funabashi 274-8510, Japan; takahasi@chem.sci.toho-u.ac.jp (M.T.); 7321201t@st.toho-u.jp (T.T.); yoshihiro.doi@sci.toho-u.ac.jp (Y.D.); 2 Institute for Quantum Medical Science, National Institutes for Quantum Science and Technology, Chiba 263-8555, Japan; cigaras2023@gmail.com (C.I.); hirocky01@nifty.com (H.M.); fukiko.hihara@gmail.com (F.H.); oikawa.masakazu@qst.go.jp (M.O.); suzuki.hisashi@qst.go.jp (H.S.); zhang.ming-rong@qst.go.jp (M.-R.Z.); higashi.tatsuya@qst.go.jp (T.H.); 3 Department of Diagnostic Radiology, Kanagawa Cancer Center, Kanagawa 241-8515, Japan; h-kurihara@kcch.jp

**Keywords:** copper requirement, cancer, PIXE, tripodal amine ligands, ^64^Cu^2+^ ions, ^64^Cu^2+^ complexes

## Abstract

Copper (Cu) is a critical element for cancer cell proliferation and considerably accumulates in the nucleus. ^64^Cu^2+^ is an anticancer radiopharmaceutical that targets the copper requirement of cancer cells. However, intravenously injected ^64^Cu^2+^ ions primarily accumulate in the liver. Ligand complexation of ^64^Cu^2+^ may be a promising method for increasing tumor delivery by reducing liver uptake. In this study, we used three tripodal amine ligands [tris(2-aminoethyl)amine (Tren), diethylenetriamine (Dien), and tris(2-pyridylmethyl)amine (TPMA)] to enclose ^64^Cu^2+^ ions and compared their in vivo tumor and liver uptakes using a tumor-bearing xenograft mouse model of the extrahepatic bile duct carcinoma cell line TFK-1. We examined intracellular Cu distribution using microparticle-induced X-ray emission (micro-PIXE) analysis of these compounds. ^64^Cu^2+^-Tren and ^64^Cu^2+^-Dien showed higher tumor uptake than ^64^Cu^2+^-TPMA and ^64^Cu^2+^ ions in TFK-1 tumors. Among the three ^64^Cu^2+^ complexes and ^64^Cu^2+^ ions, liver uptake was inversely correlated with tumor uptake. Micro-PIXE analysis showed that in vitro cellular uptake was similar to in vivo tumor uptake, and nuclear delivery was the highest for ^64^Cu^2+^-Tren. Conclusively, an inverse correlation between tumor and liver uptake was observed using three ^64^Cu^2+^ complexes of tripodal amine ligands and ^64^Cu^2+^ ions. These results provide useful information for the future development of anticancer ^64^Cu radiopharmaceuticals.

## 1. Introduction

Copper (Cu) is essential for cancer cell growth and proliferation [1,2,3]. The concentration of Cu is higher in tumor tissues and serum than in normal tissues in several types of cancer [4,5,6,7]. Cu accumulates in the nuclei of cancer cells and plays an important role in transcription-related tumor proliferation and metastasis [4,8]. Therefore, targeting the Cu requirement of cancer cells is considered a promising avenue for the development of anticancer drugs. Several studies have been conducted to investigate the potential of ^64^Cu^2+^ ions as antitumor radiotherapeutic drugs [9,10,11,12,13]. ^64^Cu is a useful radionuclide for theranostic purposes. ^64^Cu decays via β^+^ (0.653 MeV, 17.4%), β^−^ (0.574 MeV, 40%), and electron capture (42.6%); γ-ray photons from electron–positron annihilation can be used for positron emission tomography (PET) imaging, whereas β^−^ particles and Auger electrons emitted from this nuclide can be used for therapeutic purposes to damage tumor cells [14]. In particular, Auger electrons emitted from ^64^Cu demonstrate a strong capacity to damage cancer cell DNA [15]. ^64^Cu^2+^ ions uptake is high in various cancers, which inhibits tumor growth [9,16,17]. However, preclinical and human ^64^Cu^2+^ ions biodistribution studies have revealed extensive liver distribution, particularly within the first 30 min after intravenous administration, with ^64^Cu^2+^ ion levels reaching a plateau in 60–90 min and being maintained for more than 20 h [18,19]. When ^64^Cu^2+^ ions enter the blood stream, ^64^Cu^2+^ immediately binds to albumin in blood plasma, and the ^64^Cu^2+^–albumin complex is subsequently trapped in the liver [20,21]. Therefore, further studies to improve the distribution of ^64^Cu are essential for developing new anticancer drugs that utilize ^64^Cu.

**Figure 1:**
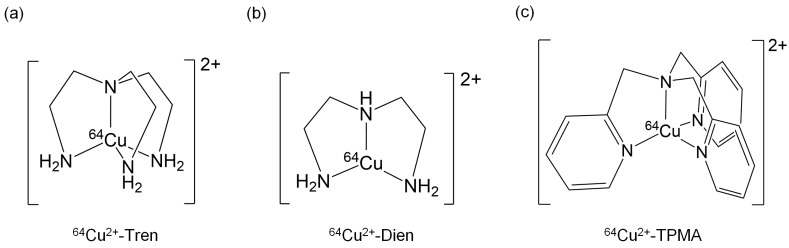
Chemical structures of (**a**) ^64^Cu^2+^-Tren, (**b**) ^64^Cu^2+^-Dien, and (**c**) ^64^Cu^2+^-TPMA.

Ligand complexation of ^64^Cu^2+^ has received attention as a potential method for decreasing liver traps and increasing tumor delivery [21,22]. Therefore, we focused on tripodal amine ligands, such as tris(2-aminoethyl)amine (Tren), diethylenetriamine (Dien), and tris(2-pyridylmethyl)amine (TPMA), and synthesized ^64^Cu^2+^-Tren, ^64^Cu^2+^-Dien, and ^64^Cu^2+^-TPMA (Figure 1). As these tripodal amine ligands can enclose ^64^Cu ions to form stable 6^4^Cu^2+^ complexes, we hypothesized that they decrease liver uptake and increase tumor uptake compared with the liver and tumor uptake of only ^64^Cu^2+^ ions.

In this study, we examined in vivo tumor and liver uptake and investigated the intracellular Cu distribution of these complexes using a tumor-bearing xenograft mouse model of an extrahepatic bile duct carcinoma cell line TFK-1 with the above three ^64^Cu^2+^ complexes. TFK-1 is a well-characterized and frequently used cell line to study extrahepatic bile duct cancer [23,24,25,26]. Bile duct carcinoma, or cholangiocarcinoma, is an aggressive tumor with a poor prognosis. The 5-year survival rate for extrahepatic bile duct cancer is 20–30% [27]. The only curative treatment for patients with extrahepatic bile duct carcinoma is surgical resection; however, the rate of resectability is low and the rate of recurrence is high [28]. Therefore, innovative drugs to treat this disease must be urgently developed. Clinical studies have reported that when ceruloplasmin, which is related to active copper metabolism, is overexpressed it is a potential prognostic marker for bile duct cancer, similar to several other cancers [29,30,31].

## 2. Results

### 2.1. Determination of the Radiochemical Purity of ^64^Cu^2+^ Complexes

**Figure 2:**
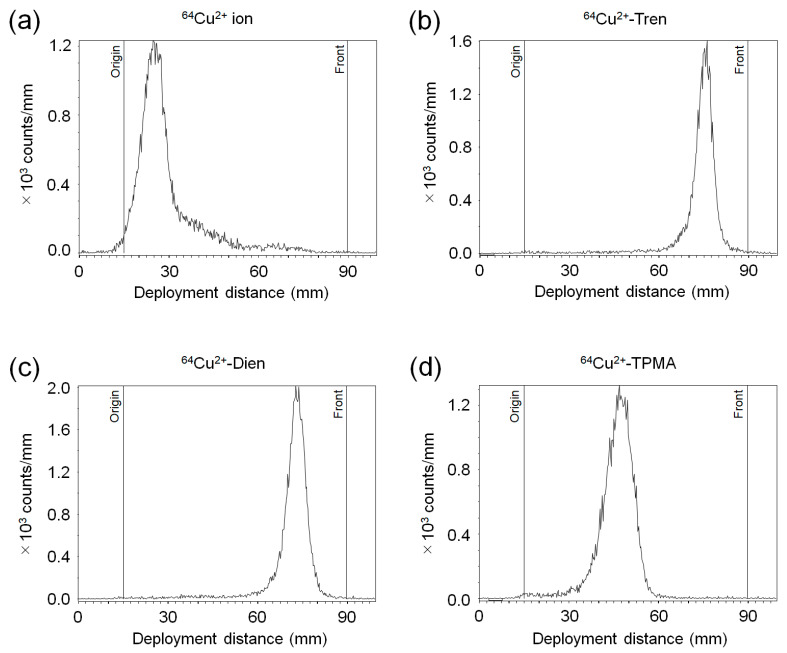
Radio-TLC (thin-layer chromatography) analysis. Representative chromatograms of (**a**) ^64^Cu^2+^ ion, (**b**) ^64^Cu^2+^-Tren, (**c**) ^64^Cu^2+^-Dien, and (**d**) ^64^Cu^2+^-TPMA.

The representative radio-chromatograms of ^64^Cu^2+^-Tren, ^64^Cu^2+^-Dien, ^64^Cu^2+^-TPMA, and ^64^Cu^2+^ ions are shown in Figure 2. ^64^Cu^2+^-Tren (*R*_f_ = 0.83), ^64^Cu^2+^-Dien (*R*_f_ = 0.78), and ^64^Cu^2+^-TPMA (*R*_f_ = 0.44) were obtained with a radiochemical purity of >95%. The *R*_f_ values of each ^64^Cu^2+^ complex were similar to those of the corresponding complexes with non-radioactive Cu^2+^.

### 2.2. In Vivo Tissue Distribution

**Figure 3:**
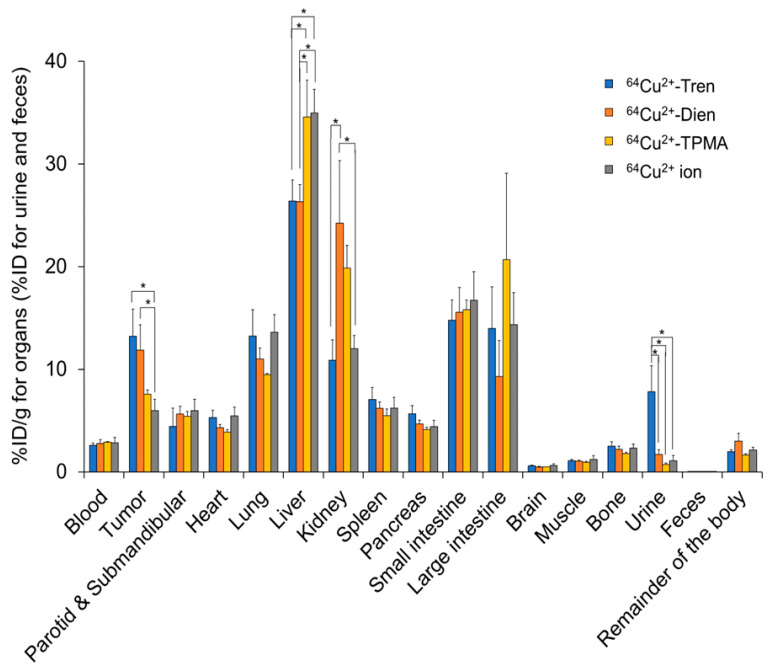
In vivo tissue distributions. Tissue distributions and excretions of Cu^2+^-Tren, Cu^2+^-Dien, Cu^2+^-TPMA, and Cu^2+^ ions in TFK-1-RFP-xenografted mice 2.5 h after injections. * Indicates significant differences (*p* < 0.05).

**Figure 4:**
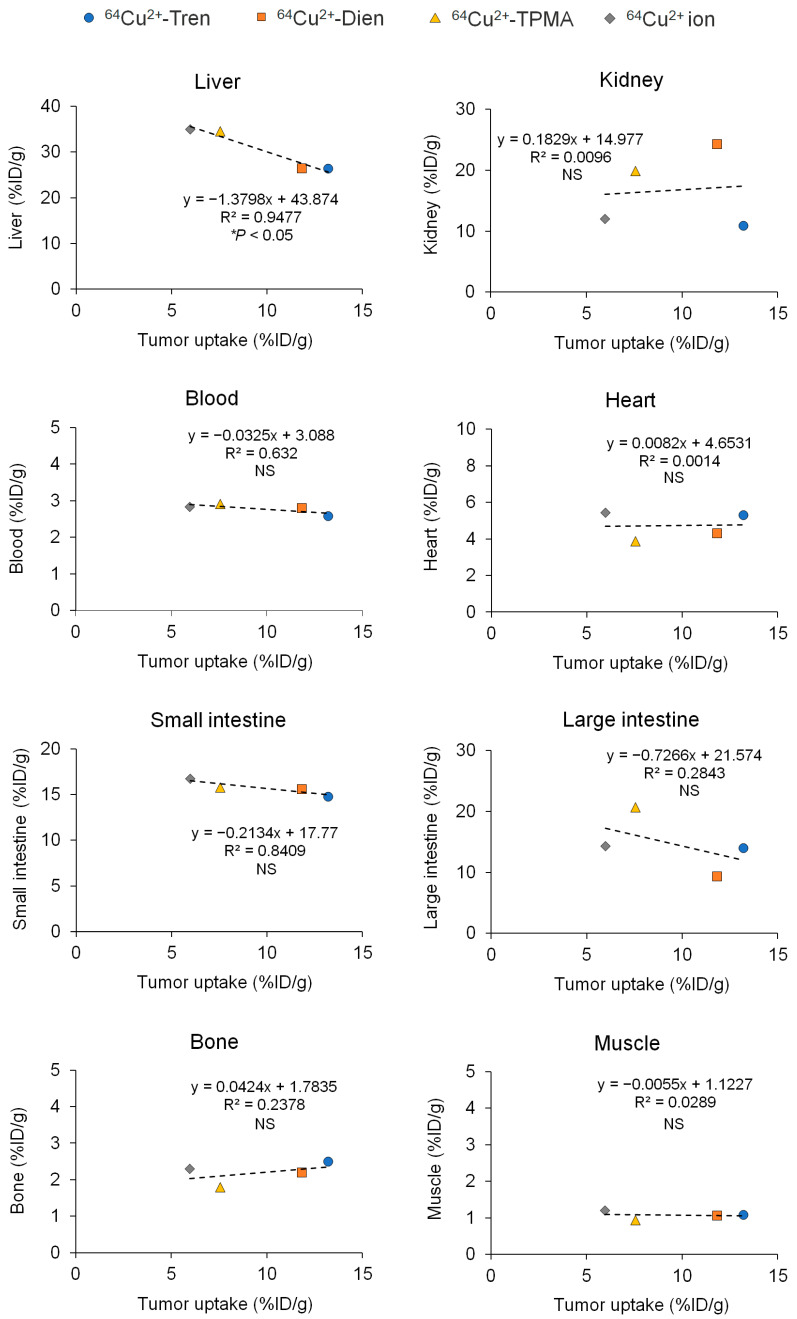
Correlations between tumor and tissue uptakes. Values of tumor and tissue uptake and excretion of Cu^2+^-Tren, Cu^2+^-Dien, Cu^2+^-TPMA, and Cu^2+^ ions in TFK-1-RFP-xenografted mice 2.5 h after injections, shown in Figure 3, are used. This figure illustrates correlations between tumors and the liver, kidney, blood, heart, small intestine, large intestine, bone, and muscle (correlations between the parotid and submandibular glands, lung, spleen, pancreas, brain, and the remainder of the body are shown in Figure S1). NS = not significant.

We compared the basic characteristics of the tissue distribution of Cu^2+^-Tren, Cu^2+^-Dien, Cu^2+^-TPMA, and Cu^2+^ in vivo. TFK-1 cells stably expressing red fluorescent protein (RFP) were used in this study (TFK-1-RFP). The tissue distribution and excretion of Cu^2+^-Tren, Cu^2+^-Dien, Cu^2+^-TPMA, and Cu^2+^ ions in mice bearing TFK-1-RFP cells 2.5 h after intravenous injections were examined (Figure 3). This time point was selected because a previous preclinical study showed that the liver and tumor distribution of Cu^2+^ ions plateaued 2 h after intravenous injection [18]. Biodistribution data are shown as the percentage of injected dose per gram (%ID/g) for organs and blood and the percentage of injected dose (%ID) for urine and feces. Cu^2+^-Tren and Cu^2+^-Dien showed higher tumor uptake than Cu^2+^-TPMA and Cu^2+^ ions (13.20 ± 2.65 %ID/g for Cu^2+^-Tren, 11.84 ± 2.47 %ID/g for Cu^2+^-Dien, 7.56 ± 0.41 %ID/g for Cu^2+^-TPMA, and 5.98 ± 1.09 %ID/g for Cu^2+^ ions) (*p* < 0.05). Cu^2+^-Tren showed a higher tumor uptake value than Cu^2+^-Dien; however, the difference was insignificant. There was no significant difference between Cu^2+^-TPMA and Cu^2+^ ions. Conversely, Cu^2+^-Tren and Cu^2+^-Dien showed lower liver uptake than Cu^2+^-TPMA and Cu^2+^ ions (26.38 ± 2.04 %ID/g for Cu^2+^-Tren, 26.33 ± 1.66 %ID/g for Cu^2+^-Dien, 34.58 ± 3.58 %ID/g for Cu^2+^-TPMA, and 34.96 ± 2.32 %ID/g for Cu^2+^ ions) (*p* < 0.05). There were no significant differences in liver uptake between Cu^2+^-Tren and Cu^2+^-Dien or between Cu^2+^-TPMA and Cu^2+^ ions. Figure 4 and Figure S1 show the correlation between tumor and tissue uptake. There was a significantly strong inverse correlation between tumor uptake and liver uptake (*R* = −0.9735, *p* < 0.05), whereas no correlation was observed in any of the other examined tissues (kidney, blood, heart, small intestine, large intestine, bone, muscle, parotid and submandibular, lung, spleen, pancreas, brain, and the remainder of the body). Of the two Cu^2+^ complexes (Cu^2+^-Tren and Cu^2+^-Dien) that showed high tumor uptake, Cu^2+^-Tren showed lower kidney uptake than Cu^2+^-Dien (10.87 ± 1.98%ID/g for Cu^2+^-Tren and 24.23 ± 6.09 %ID/g for Cu^2+^-Dien) (*p* < 0.05) and higher urinary excretion than Cu^2+^-Dien (7.81 ± 2.54 %ID for Cu^2+^-Tren and 1.68 ± 0.46 %ID for Cu^2+^-Dien) (*p* < 0.05).

### 2.3. In Silico Log P_o/w_ and Log S Studies of Cu^2+^ Complexes

As results in Figure 3 and Figure 4 indicate a tendency of liver uptake for the studied ^64^Cu^2+^ complexes, a comparison of their physicochemical properties was performed. The octanol–water partition coefficients (log *P*_o/w_) of the Cu^2+^ complexes were calculated using SwissADME software (http://www.swissadme.ch) [32]. SwissADME is a free web tool operated by the Swiss Institute of Bioinformatics (SIB), which provides predictive models for physicochemical properties, pharmacokinetics, drug compatibility, and medicinal chemistry compatibility [32]. The consensus log *P*_o/w_ (log *P*_o/w_), which is the arithmetic mean of values predicted by five lipophilicity prediction models, was used in this study.

The calculated log *P*_o/w_ values for Cu^2+^-Tren, Cu^2+^-Dien, and Cu^2+^-TPMA were −0.98, −0.52, and 0.99, respectively (Table 1). The same software was also used to calculate the water solubility log *S* (mol/L) using the estimated solubility model (ESOL) [33]. The calculated log *S* values for Cu^2+^-Tren, Cu^2+^-Dien, and Cu^2+^-TPMA were −1.91, −1.92, and −5.46, respectively (Table 1).

### 2.4. Intracellular Cu Distribution

**Figure 5:**
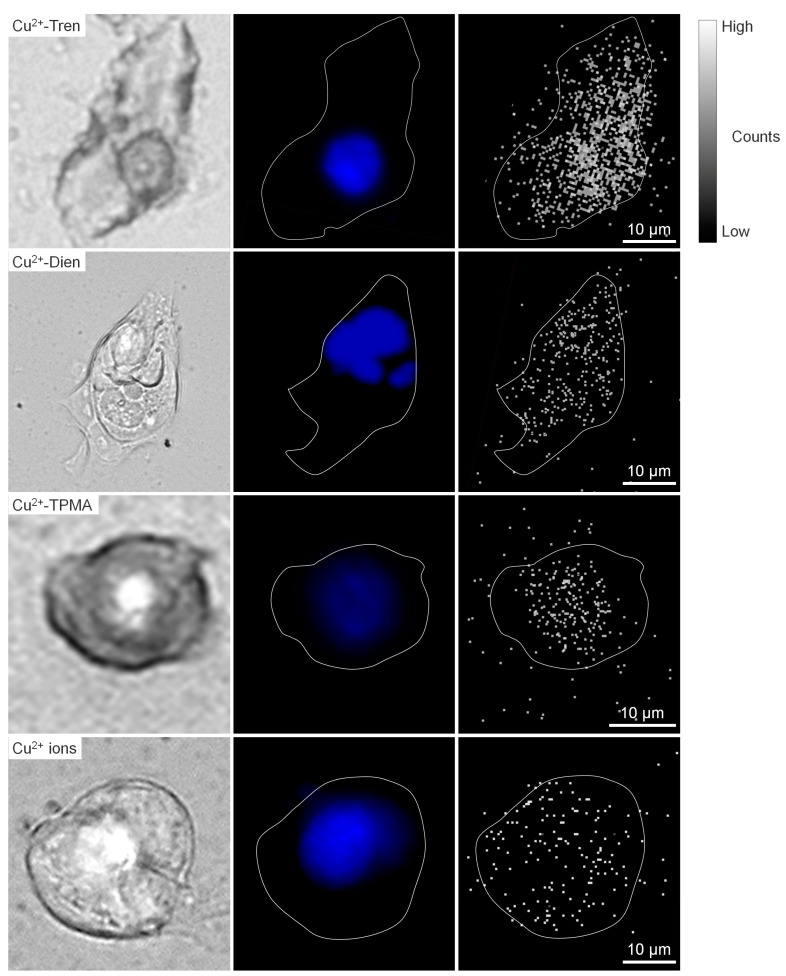
Micro-PIXE analysis. Representative images of Cu^2+^-Tren, Cu^2+^-Dien, Cu^2+^-TPMA, and Cu^2+^ ions are shown: bright field (**left** column), fluorescence from DAPI (**middle** column), and micro-PIXE (**right** column) images.

**Figure 6:**
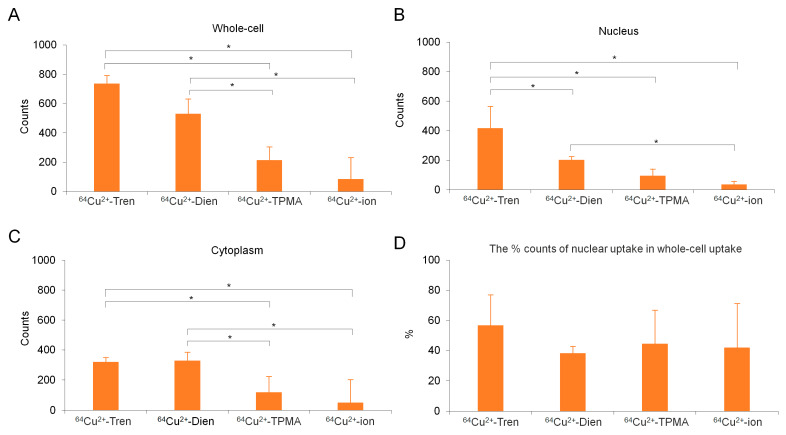
Quantitative analysis of micro-PIXE. (**A**) In vitro whole-cell, (**B**) nuclear, and (**C**) cytoplasmic uptakes of Cu^2+^-Tren, Cu^2+^-Dien, Cu^2+^-TPMA, and Cu^2+^ ions are shown. (**D**) The percentage of nuclear uptake to whole-cell uptake is also shown. * Indicates significant differences (*p* < 0.05).

Micro-particle-induced X-ray emission (micro-PIXE) analysis was performed to investigate in vitro whole-cell, nuclear, and cytoplasmic uptakes of Cu^2+^-Tren, Cu^2+^-Dien, Cu^2+^-TPMA, and Cu^2+^ ions at a 1 µm spatial resolution (Figure 5 and Figure 6). The in vitro whole-cell uptake showed a parallel tendency to the in vivo tumor uptake; Cu^2+^-Tren and Cu^2+^-Dien showed higher uptakes than Cu^2+^-TPMA and Cu^2+^ ions (736.00 counts ± 156.03 for Cu^2+^-Tren, 530.60 counts ± 106.87 for Cu^2+^-Dien, 118.58 counts ± 59.33 for Cu^2+^-TPMA, and 49.43 counts ± 34.33 for Cu^2+^ ions) (*p* < 0.05). A positive correlation was observed between in vitro whole-cell uptake and in vivo tumor uptake (*R* = 0.992, *p* = 0.00772) (Figure S2). Similarly, we observed a consistent correlation between in vitro nuclear uptake and in vivo tumor uptake (417.6 counts ± 150.02 for Cu^2+^-Tren, 203.00 counts ± 25.13 for Cu^2+^-Dien, 94.82 counts ± 48.07 for Cu^2+^-TPMA, and 35.57 counts ± 25.02 for Cu^2+^ ions) (*p* < 0.05). Notably, among the three Cu^2+^ complexes and Cu^2+^ ions, Cu^2+^-Tren showed the highest average value in percentage counts of nuclear uptake/whole-cell uptake, although there were no significant differences.

## 3. Discussion

^64^Cu^2+^ ions are promising as an anticancer therapeutic drug targeting the copper requirement of cancer cells; however, intravenously injected ^64^Cu^2+^ ions primarily accumulate in the liver. Complexation of the ligand with ^64^Cu^2+^ may be a potential way to increase delivery to the tumor by decreasing liver uptake.

In this study, we compared in vivo tumor and liver uptakes of three ^64^Cu^2+^ complexes with tripodal amine ligands ^64^Cu^2+^-Tren, ^64^Cu^2+^-Dien, and ^64^Cu^2+^-TPMA with those of ^64^Cu^2+^ ions using a tumor-bearing xenograft mouse model of the extrahepatic bile duct carcinoma cell line TFK-1. In vivo tissue distribution showed that ^64^Cu^2+^-Tren and ^64^Cu^2+^-Dien showed higher tumor uptake and lower liver uptake than ^64^Cu^2+^-TPMA and ^64^Cu^2+^ ions. In addition, we observed a parallel trend between in vitro cellular uptake and in vivo tumor uptake, with ^64^Cu^2+^-Tren exhibiting high whole-cell and nuclear uptake in TFK-1 cells.

Copper is an essential element for cancer cell growth and proliferation [1]; therefore, ^64^Cu^2+^ ions have been extensively investigated as a potential antitumor radioactive therapeutic drug [9]. However, ^64^Cu^2+^ ions were widely distributed in the liver immediately after intravenous administration [18,19]. Therefore, improving the distribution of ^64^Cu^2+^ ions is important for the development of novel ^64^Cu anti-cancer drugs. This study demonstrated the potential of ^64^Cu^2+^-Tren and ^64^Cu^2+^-Dien to improve ^64^Cu^2+^ delivery to tumors by reducing liver traps. Consequently, these two ^64^Cu^2+^ complexes emerged as promising candidates for further development. Cu is highly concentrated in tumor cells and tissues [4] and accumulates in the nuclei of cancer cells [4,8]. This study demonstrated that Cu^2+^-Tren and Cu^2+^-Dien showed higher whole-cell and nuclear uptakes than Cu^2+^-TPMA and Cu^2+^ ions did. Consequently, ^64^Cu^2+^-Tren and ^64^Cu^2+^-Dien are good candidates among the examined Cu^2+^ complexes and Cu^2+^ ions. Cu^2+^-Tren showed much higher in vitro nuclear uptake and (although there were no significant differences) a higher tendency for in vivo tumor uptake, in vitro whole-cell uptake, and percentage counts of nuclear uptake in the whole cell than ^64^Cu^2+^-Dien did. Cu^2+^-Tren showed rapid urinary excretion, whereas ^64^Cu^2+^-Dien showed kidney retention at the examined time points. Based on these observations, Cu^2+^-Tren is a better alternative than ^64^Cu^2+^-Dien.

Notably, we found a significantly strong inverse correlation between tumor uptake and liver uptake; however, no correlation was observed in any of the other examined tissues (Figure 4 and Figure S1) for ^64^Cu^2+^-Tren, ^64^Cu^2+^-Dien, ^64^Cu^2+^-TPMA, and ^64^Cu^2+^ ions. Previous studies have shown that intravenously administered ^64^Cu^2+^ ions immediately bind to albumin in the blood plasma, and the ^64^Cu^2+^-albumin complex is subsequently trapped in the liver [20,21]. Ligand complexation of ^64^Cu^2+^ can be a promising strategy to reduce liver traps and facilitate tumor delivery [21,22]. In addition, lipophilicity is an important factor in determining excretion [34,35,36]. Therefore, the present study focused on three ^64^Cu^2+^ complexes of tripodal amine ligands with different lipophilicities, log *P*_o/w_. Log *P*_o/w_ is an indicator of lipophilicity, and the calculated log *P*_o/w_ values for Cu^2+^-Tren, Cu^2+^-Dien, and Cu-TPMA were −0.98, −0.52, and 0.99, respectively. A previous study reported that if a log *P*_o/w_ value is less than 0, the compound is classified as hydrophilic, and if the value is greater than 0, the compound is classified as lipophilic [37,38]. In addition, hydrophilic compounds increase the proportion of urinary excretion via the kidney, and lipophilic compounds are likely to show liver excretion [39,40]. Therefore, based on this knowledge and our results, it is considered reasonable that ^64^Cu^2+^-Tren and ^64^Cu^2+^-Dien with log *P*_o/w_ < 0 (hydrophilic) increased the proportion of urinary excretion via the kidney, whereas Cu^2+^-TPMA with a log *P*_o/w_ > 0 (lipophilic) showed liver excretion.

Water solubility is one of the critical factors in achieving the desired drug concentration in systemic circulation to achieve the required pharmacological response [41]. To successfully develop intravenous formulations, water solubility must be high to deliver sufficient quantities of the active ingredient through a limited drug dosage [42]. The calculated log *S* (mol/L) values for Cu^2+^-Tren, Cu^2+^-Dien, and Cu^2+^-TPMA were −1.91, −1.92, and −5.46, respectively, suggesting that ^64^Cu^2+^-Tren and ^64^Cu^2+^-Dien are highly soluble and have potential as intravenous preparations.

This study had several limitations. First, this study aimed to perform a basic comparative study of ^64^Cu complexes with tripodal amine ligands and used only one cell line of extrahepatic bile duct carcinoma. In future studies, it will be beneficial to use other cell lines of bile duct carcinoma and different types of cancer. Second, this study demonstrated that ^64^Cu^2+^-Tren and ^64^Cu^2+^-Dien showed higher in vivo and in vitro tumor uptake in TFK-1 cells. Further studies of their detailed biodistribution over time and in vitro and in vivo therapeutic efficacy are warranted for the future development of ^64^Cu^2+^-Tren and ^64^Cu^2+^-Dien. Finally, we did not focus on the transport mechanisms of Cu^2+^ complexes in this study. Previous studies of the cytotoxicity of several copper(II) complexes for cancer treatment have demonstrated that copper transporter 1 plays critical roles in cancer cell uptake of copper(II) complexes [43]. Therefore, copper transporter 1 may also contribute to the transportation of ^64^Cu^2+^-Tren and ^64^Cu^2+^-Dien. Elucidation of the transport mechanism of these Cu^2+^ complexes is critical for future developmental studies of these compounds.

## 4. Materials and Methods

### 4.1. Reagents and Materials

Ligands Tren, Dien, and TPMA were obtained from Tokyo Chemical Industry Co., Ltd. (Tokyo, Japan). Copper(II) chloride dihydrate and ammonium acetate were of guaranteed reagent grade and were obtained from Fujifilm Wako Pure Chemical Corporation (Osaka, Japan). Copper(II) perchlorate was purchased from Nacalai Tesque, Inc. (Kyoto, Japan). Ultrapure water was purchased from Kanto Chemical Co. (Tokyo, Japan). All reagents and solvents were used without further purification. The Cu^2+^-Tren, Cu^2+^-Dien, and Cu^2+^-TPMA used for TLC analysis were prepared as previously described in the literature [44,45], and complexes used in the micro-PIXE analysis were also synthesized as previously described [46,47,48].

### 4.2. Preparation of ^64^Cu^2+^ Complexes

^64^CuCl_2_ (in 0.05N HCl) was obtained from PDR Pharma (Tokyo, Japan). ^64^CuCl_2_ solution was evaporated to dryness and dissolved in water. ^64^Cu^2+^-Tren, ^64^Cu^2+^-Dien, and ^64^Cu^2+^-TPMA (10 MBq/21 nmol) were synthesized by adding a ^64^Cu^2+^ aqueous solution (10 MBq/50 µL) to each ligand aqueous solution (21 nmoL/50 µL). To determine the radiochemical purity of prepared ^64^Cu^2+^ complexes, 1 µL of each sample solution was spotted on an HPTLC NH_2_ Silica Gel 60 F_254_ glass plate (Fujifilm Wako Pure Chemical Corporation, Osaka, Japan) and developed using aqueous ammonium acetate solution (0.5 M). Radioactivity on the HPTLC plates was measured using a radio-TLC system (Raytest PET MiniGITA Star; Elysia S.A., Liège, Belgium).

### 4.3. Cell Line and Culture

The human extrahepatic bile duct carcinoma cell line TFK-1 was obtained from the RIKEN Bioresource Research Center (Ibaraki, Japan) and immediately expanded and frozen in our laboratory. For animal experiments, TFK-1 cells stably expressing red fluorescent protein (RFP) (TFK-1-RFP) were used. To establish the TFK-1-RFP cell line, TFK-1 cells were transfected with RFP lentivirus (Lenti-Labeler Cell Labeling System, System Biosciences, Palo Alto, CA, USA) following the manufacturer’s protocol. A clone strongly expressing RFP was selected by limiting dilution and was denoted as TFK-1-RFP. Early passage TFK-1 and TFK-1-RFP cells, with <2–3 months of cumulative subculture, were used for all experiments. Cells were grown in RPMI-1640 medium (Invitrogen, Carlsbad, CA, USA) supplemented with 10% fetal bovine serum (Gibco, Grand Island, NY, USA) and incubated at 37 °C in a humidified atmosphere of 5% CO_2_. Exponentially growing cells were detached from plates using trypsin (0.5 *w*/*v*% Trypsin-5.3 mmol/L EDTA·4Na Solution without Phenol Red (×10), Fujifilm Wako Pure Chemical Corporation, Osaka, Japan), and the number of viable cells was determined using the trypan blue dye (Bio-Rad, Hercules CA, USA) exclusion method.

### 4.4. Animal Model

Animal experimental procedures were approved by the Animal Ethics Committee of the National Institutes for Quantum Science and Technology (QST, Chiba, Japan) and conducted following institutional guidelines. SCID beige mice (7-week-old females) were obtained from Charles River Laboratories Japan (Yokohama, Japan) and used for in vivo biodistribution experiments. TFK-1-RFP cells (5 × 10^6^ cells) suspended in RPMI-1640 were pre-mixed with Matrigel at a ratio of 50:50 (*v*/*v*) and subcutaneously injected into the flanks of the mice. Mice bearing tumors of approximately 5 mm in diameter were used to examine tissue distribution in vivo.

### 4.5. Tissue Distribution In Vivo

To compare tumor and liver uptakes of ^64^Cu^2+^ complexes (^64^Cu^2+^-Tren, ^64^Cu^2+^-Dien, and ^64^Cu^2+^-TPMA) and ^64^Cu ions, in vivo tissue distribution was investigated in mice bearing TFK-1-RFP cells. Mice were administered 1.85 MBq of ^64^Cu^2+^-Tren, ^64^Cu^2+^-Dien, ^64^Cu^2+^-TPMA, and ^64^Cu^2+^ ions intravenously (*n* = 3–4/group) and sacrificed at 2.5 h to collect tissues (parotid, submandibular, heart, lung, liver, kidney, spleen, pancreas, small intestine, large intestine, brain, muscle, bone, tumor, and the remainder of the body) and blood. Urine and feces were collected during the 2.5 h post-administration using polyethylene-laminated filter paper. Radioactivity levels were counted using a γ-counter (1480 Automatic gamma counter Wizard 3; PerkinElmer Inc., Waltham, MA, USA). The %ID/g for organs and blood and the %ID for urine and feces were calculated.

### 4.6. In Silico Log P_o/w_ and Log S Studies of ^64^Cu^2+^ Tripodal Amine Complexes

The log *P*_o/w_ values of the Cu^2+^ complexes were calculated using SwissADME software [32]. SwissADME free web tools provide predictive models for physicochemical properties, pharmacokinetics, drug compatibility, and medicinal chemistry compatibility. Water solubility (log *S*), one of the most important physicochemical properties of drugs, was also calculated using the same software.

### 4.7. Micro-PIXE Analysis

A micro-PIXE analysis was carried out at the QST Electrostatic Accelerator Facility [49]. The system consists of a 3.0 MeV proton microbeam (ϕ = 1 μm) combined with a 1.7 MV tandem accelerator and an ion source. Cells were detached from culture plates using trypsin to make cell suspensions of 8.0 × 10^4^ cells/mL in RPMI-1640 medium. Cell suspensions were dropped on a 5 μm Mylar film (Chemplex Inc., Palm City, FL, USA) in a culture dish and incubated at 37 °C in a humidified atmosphere containing 5% CO_2_ for 2 d. Cells were incubated in a medium with 1 mM non-radioactive compounds (Cu^2+^-Tren, Cu^2+^-Dien, Cu^2+^-TPMA, and Cu^2+^ ions) for 4 h. Thereafter, cells were washed twice with phosphate-buffered saline, fixed with 4% paraformaldehyde (Fujifilm Wako Pure Chemical Corporation, Osaka, Japan), and rinsed three times with 150 mM ammonium acetate buffer. Cells were subsequently stained with DAPI (4′,6-diamidino-2-phenylindole; Bio-Rad, Hercules, CA, USA) for nuclear localization and observed under a fluorescence microscope (BZ-X810, Keyence, Osaka, Japan). Samples were air-dried at room temperature for more than 24 h and subjected to micro-PIXE analysis [49]. The sample preparation procedure was based on the previous literature with modifications [50]. Distributions of Cu, P, K, Ca, Al, Fe, and S in each group were determined using Kα lines. Each micro-PIXE image was obtained for five cells per group. The cell morphology in micro-PIXE analysis was determined by matching micro-PIXE images of phosphorus and potassium, which are distributed uniformly throughout cells. Nuclei localization was determined via fluorescence microscope images of DAPI staining. Cu signals in the nucleus, cytoplasm, and whole cells were counted.

### 4.8. Statistical Analysis

Data were expressed as the mean ± SD. Multiple comparisons were conducted using parametric one-way analysis of variance with the Tukey−Kramer post-hoc test. All statistical analyses were conducted at a significance level of *p* < 0.05. Data analyses were performed using JMP 13.2.0 (SAS Institute, Cary, NC, USA).

## 5. Conclusions

This study demonstrated that ^64^Cu^2+^-Tren and ^64^Cu^2+^-Dien showed higher in vivo tumor uptake and in vitro cellular and nuclear uptake than ^64^Cu^2+^-TPMA and ^64^Cu^2+^ ions in TFK-1 cells. In the in vivo study, an inverse correlation was observed between tumor and liver uptake among the three examined ^64^Cu^2+^ complexes and ^64^Cu^2+^ ions. ^64^Cu^2+^-Tren and ^64^Cu^2+^-Dien are promising candidates for the development of anticancer ^64^Cu drugs. This study provides useful information for the future development of ^64^Cu radiopharmaceuticals.

## Figures and Tables

**Table 1:** Basic physicochemical properties of Cu^2+^ complexes in this study.

Complex	Molecular Formula	Molecular Weight	Consensus Log *P*_o/w_	Log *S*
Cu(Tren)Cl_2_	C_6_H_18_Cl_2_CuN_4_	280.69	–0.98	–1.91
Cu(Dien)Cl_2_	C_4_H_13_Cl_2_CuN_3_	237.62	–0.52	–1.92
Cu(TPMA)Cl_2_	C_18_H_18_Cl_2_CuN_4_	424.81	0.99	–5.46

## Data Availability

Data are within the article and Supplementary Materials.

## Supplementary Materials

The following supporting information can be downloaded at: https://www.mdpi.com/article/10.3390/ph17070820/s1, Figure S1: Correlation between tumor uptake and tissue uptake. Figure S2: Comparison of in vivo tumor uptake and in vitro whole-cell uptake.
