# Supplementary material for: 64Cu2+ Complexes of Tripodal Amine Ligands’ In Vivo Tumor and Liver Uptakes and Intracellular Cu Distribution in the Extrahepatic Bile Duct Carcinoma Cell Line TFK-1: A Basic Comparative Study
Source: Pharmaceuticals (Basel). 2024 Jun 21;17(7):820. doi: 10.3390/ph17070820 (PMC11280065; doi:10.3390/ph17070820)
Supplement: Supplementary file 1 [file pharmaceuticals-17-00820-s001.zip › pharmaceuticals-3056932-supplementary.pdf]

Supplementary Materials: $^{64}Cu^{2+}$ complexes of Tripodal Amine Ligands on In Vivo Tumor and Liver Uptake and Intracellular Cu distribution in the Extrahepatic Bile Duct Carcinoma Cell Line TFK-1: A Basic Comparative Study

Mitsuhiro Shinada, Masashi Takahashi, Chika Igarashi, Hiroki Matsumoto, Fukiko Hihara, Tomoko Tachibana, Masakazu Oikawa, Hisashi Suzuki, Ming-Rong Zhang, Tatsuya Higashi, Hiroaki Kurihara, Yukie Yoshii, Yoshihiro Doi

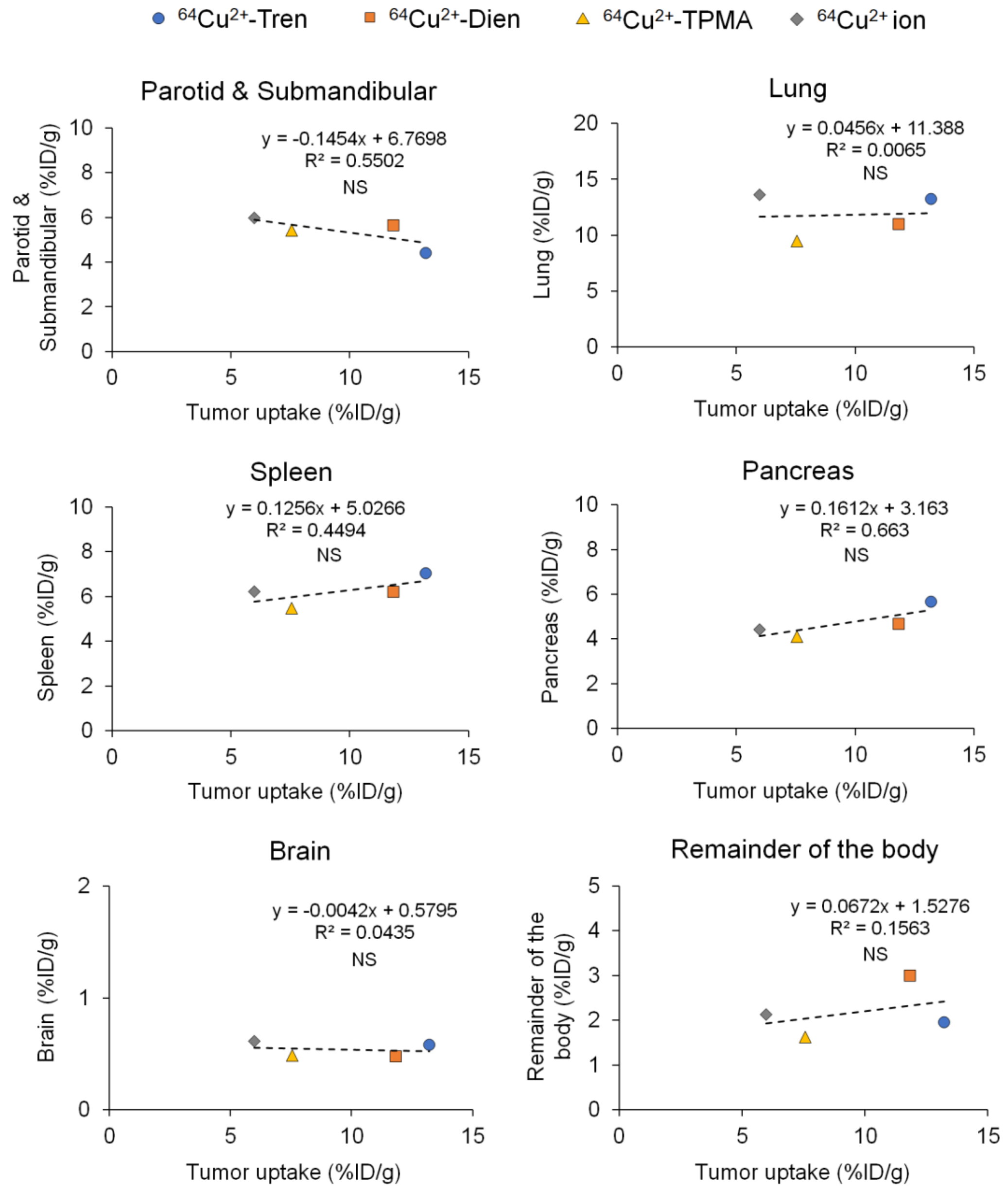

Figure S1. Correlation between tumor uptake and tissue uptake. Values of tumor uptake and tissue uptake and excretion of Cu^{2+}-Tren, Cu^{2+}-Dien, Cu^{2+}-TPMA, and Cu^{2+} ion in TFK-1-RFP-xenografted mice at 2.5 h after injections are used. This figure shows correlation for parotid and submandibular, lung, spleen, pancreas, brain, and remainder of the body. (That for liver, kidney, blood, heart, small intestine, large intestine, bone, and muscle is shown in Fig. 4).

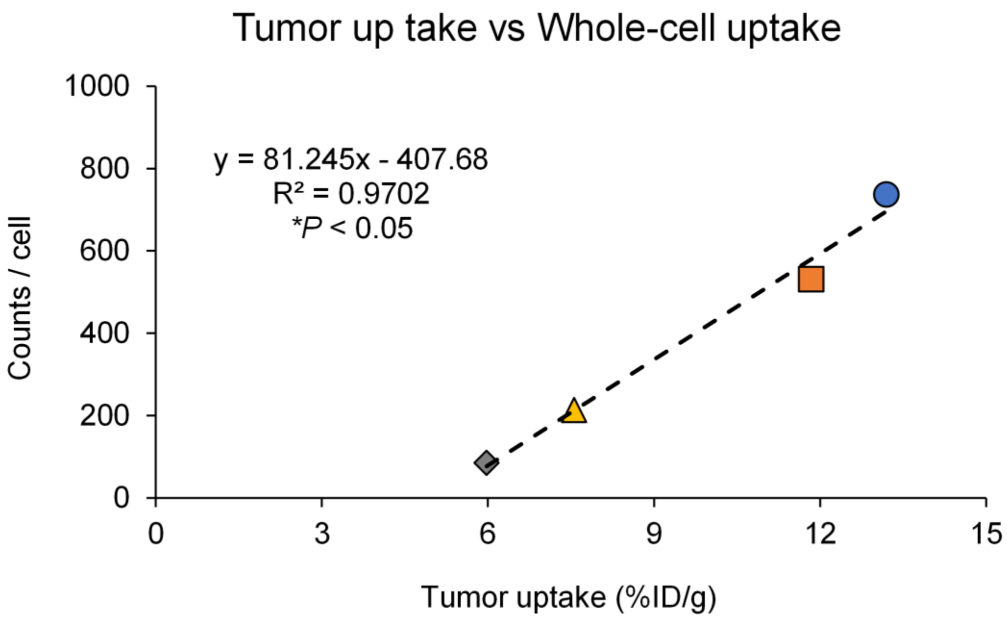

Figure S2. Comparison of *in vivo* tumor uptake and *in vitro* whole-cell uptake. Scatter plot showing a significantly positive correlation between *in vivo* tumor uptake and *in vitro* whole-cell uptake ($R^2 = 0.970$, $P < 0.05$).
